# The methylome of the celiac intestinal epithelium harbours genotype-independent alterations in the HLA region

**DOI:** 10.1038/s41598-018-37746-6

**Published:** 2019-02-04

**Authors:** Nora Fernandez-Jimenez, Koldo Garcia-Etxebarria, Leticia Plaza-Izurieta, Irati Romero-Garmendia, Amaia Jauregi-Miguel, Maria Legarda, Szilvia Ecsedi, Ainara Castellanos-Rubio, Vincent Cahais, Cyrille Cuenin, Davide Degli Esposti, Iñaki Irastorza, Hector Hernandez-Vargas, Zdenko Herceg, Jose Ramon Bilbao

**Affiliations:** 1 Epigenetics Group, International Agency for Research on Cancer (IARC), 69372 Lyon CEDEX 08, Lyon, France; 2 Department of Genetics, Physical Anthropology and Animal Physiology, University of the Basque Country (UPV/EHU), Biocruces-Bizkaia Health Research Institute, Leioa, Basque Country 48940 Spain; 3 Pediatric Gastroenterology Unit, Cruces University Hospital, Barakaldo, Basque Country 48903 Spain; 4 Spanish Biomedical Research Center in Diabetes and associated Metabolic Disorders (CIBERDEM), Madrid, Spain; 5 Present Address: Department of Gastrointestinal and Liver Diseases, Biodonostia Health Research Institute, Donostia, Basque Country Spain; 6 Present Address: Universite Côte d’Azur, INSERM, CNRS, iBV, Nice, France; 7 Present Address: Irstea - Laboratoire d’écotoxicologie, UR “Milieux aquatiques, écologie et pollutions”, Villeurbanne, France; 8 Present Address: Department of Immunology, Virology and Inflammation; TGF beta and Immune Evasion Group; Cancer Research Center of Lyon; INSERM, CNRS, Centre Léon Bérard Hospital, Lyon, France

## Abstract

The Human Leucocyte Antigen (HLA) *locus* and other DNA sequence variants identified in Genome-Wide Association (GWA) studies explain around 50% of the heritability of celiac disease (CD). However, the pathogenesis of CD could be driven by other layers of genomic information independent from sequence variation, such as DNA methylation, and it is possible that allele-specific methylation explains part of the SNP associations. Since the DNA methylation landscape is expected to be different among cell types, we analyzed the methylome of the epithelial and immune cell populations of duodenal biopsies in CD patients and controls separately. We found a cell type-specific methylation signature that includes genes mapping to the HLA region, namely *TAP1* and *HLA-B*. We also performed Immunochip SNP genotyping of the same samples and interrogated the expression of some of the affected genes. Our analysis revealed that the epithelial methylome is characterized by the loss of CpG island (CGI) boundaries, often associated to altered gene expression, and by the increased variability of the methylation across the samples. The overlap between differentially methylated positions (DMPs) and CD-associated SNPs or variants contributing to methylation quantitative trait *loci* (mQTLs) is minimal. In contrast, there is a notable enrichment of mQTLs among the most significant CD-associated SNPs. Our results support the notion that DNA methylation alterations constitute a genotype-independent event and confirm its role in the HLA region (apart from the well-known, DQ allele-specific effect). Finally, we find that a fraction of the CD-associated variants could exert its phenotypic effect through DNA methylation.

## Introduction

Celiac disease (CD [MIM 212750]) is a common, immune-mediated enteropathy that develops in genetically predisposed individuals upon exposure to dietary gluten. HLA-DQ2 and HLA-DQ8 haplotypes account for around 40% of the genetic contribution to CD and are present in virtually all celiac patients. The majority of efforts aimed at identifying the genetic predisposition to the disease have relied on SNP association studies, and the contribution of common genetic variation identified so far, including HLA, amounts for roughly 50% of the heritability^1^. However, other layers of genomic information that are independent from DNA sequence variation could also contribute to the pathogenesis of CD but have been left unscrutinized. In this sense, the identification of changes in DNA methylation that could have marked susceptible genomes in response to early-life environmental exposures, could help discover novel genomic regions involved in the onset and development of CD.

In the last decade, evidence has been accumulated suggesting that epigenetic makeup may represent an important causal contributor to phenotypic variation and common diseases. For example, several immune diseases, including inflammatory bowel disease (IBD), type 1 diabetes (T1D) and different cancers, have been shown to harbour disease-specific genome-wide DNA methylation signatures^2–4^. However, there is growing criticism to the way this kind of studies have been carried out and recent publications strongly recommend (a) taking special care on cell type sorting, (b) controlling for genotype-effects on methylation profiles and (c) assesing the functional impact of the observed changes by transcription analyses^5,6^. Moreover, other approaches apart from the sole comparison of average methylation levels of single CpG positions in case/control studies should be implemented, such as accounting for the differential variation between groups to identify relevant methylation changes or field defects in the case of cancer^6^.

In CD, a previous candidate-gene methylation analysis in duodenal biopsies of patients was able to detect changes in the promoters of several NFkB-related genes^7^. However, one must consider that in the normal duodenum, the proportion of intraepithelial lymphocytes (IELs) does not exceed 5–10 IELs per 100 epithelial cells, while values in active CD mucosa largely exceed the pathological threshold of 20–25%^8^. These two cell compartments (epithelial and immune) are the two main fractions in the small intestinal mucosa and are expected to harbor different methylation profiles characteristic of each cell type. Analysis of DNA methylation in whole tissue will inevitably lead to misleading results because the different immune:epithelial cell type proportions in CD and control tissues will mask the disease-related changes in each cell group. For this reason, in this study we have performed a separate genome-wide methylation profiling in each of the two main cell populations from the duodenal mucosa.

There is also a growing interest in SNPs that may have influence on the methylation levels of nearby CpG sites, known as methylation Quantitative Trait *Loci* (mQTLs). It has been shown that the proportion of mQTLs was higher in the top associated SNPs from a bipolar disorder GWAS than in a random set of SNPs with comparable allele frequencies, and that those SNPs are located in microRNA binding sites^9^. More recently, the scrutiny of differentially methylated CpGs identified in schizophrenia patients revealed an enrichment for genes related to development and neurodifferentiation as well as an overrepresentation of GWAS risk *loci*^10^. Finally, allele-specific methylation has been reported in certain disease-associated SNPs, including CD, suggesting that DNA methylation could explain the phenotypic effects of at least some of the SNPs predisposing to CD^11^. Therefore, to be able to determine the scale at which differential DNA methylation is mediating SNP associations, it is important to have dense SNP genotype data available from the same individuals where the methylome study has been performed.

The aim of the present study was to define the duodenal cell population-specific methylome of CD, to understand its interaction with the CD-associated genetic variation and to explore the putative consequences of the identified methylation changes at the transcriptional level. Additionally, we aimed to ascertain whether a fraction of the genetic association in CD could be explained by allele-specific DNA methylation.

## Methods

### Subjects and samples

The study was approved by the Cruces University Hospital and Basque Clinical Trials and Ethics Committees (codes CEIC-E13/20 and PI2013072, respectively) and informed consent was obtained from all subjects or their parents. All experiments were performed in accordance with the relevant guidelines and regulations. CD was diagnosed according to the ESPGHAN (European Society of Pediatric Gastroenterology Hepatology and Nutrition) criteria in force at the time of recruitment, including anti-transglutaminase antibody (TGA) determinations as well as a confirmatory small bowel biopsy. Biopsy specimens from the distal duodenum were obtained during routine endoscopy from CD patients at the time of diagnosis (CD patients with clinically active disease, positive for autoantibodies againts tissue transglutaminase and presenting with atrophy of intestinal *villi* and crypt hyperplasia, who were on a non-restricted gluten-containing diet at that time), treated patients after at least two years on GFD (asymptomatic, autoantibody-negative and a normalized intestinal mucosa) and from non-celiac controls that underwent endoscopy for causes other than inflammation (Table S1). The complete biopsies used for biological validation were snap-frozen and stored in liquid nitrogen until nucleic acid extraction. Those biopsies from which immune and epithelial cell populations were sorted, were processed immediately as described below. Biopsies from cases and controls were stored in the same way for each of the experiments and all comparisons were performed between samples treated in the same way.

### Sample processing, cell separation and purification of nucleic acids

Enterocytes and immune cells are the principal cell populations of the duodenal mucosa. The former express the epithelial cell adhesion molecule EpCAM (CD326) on their surface, while leukocytes harbor the CD45 antigen encoded by the *PTPRC* gene. These characteristics make it possible to separate and distinguish both cell populations from a biopsy sample, and allow the independent study of both cell types in CD. For that purpose, MACS magnetic cell separation technology was used (except when otherwise stated, all components from Miltenyi Biotec, Bergisch Gladbach, Germany), following the manufacturer’s protocol. Briefly, cells were mechanically separated from fresh biopsies by rotation-agitation in 10 ml of RPMI medium supplemented with 2% FBS, 1% DTT and 5 nM EDTA for 1 hour. The *lamina propria* and *debris* were removed by filtration through 30 µm pre-separation filters (cat. no. 130-041-407) and dead cell removal kit (cat. no. 130-090-101) was used to prepare a viable single-cell homogenous suspension. Live cells were labelled with CD45 magnetic microbeads (cat. no. 130-045-801) in the presence of FcR blocking reagent (cat. no. 130-059-901) to increase the specificity of antibody labelling. The antibody-labelled cell-suspension was applied to a magnetic separation column (cat. no. 130-042-201) and unlabelled CD45- cells (mainly the epithelial CD326+ fraction) were collected with the flow-through, while CD45+ cells were recovered after removing the column from the magnet.

Both cell fractions and the *lamina propria* were stored in RLT lysis buffer (Qiagen, Hilden, Germany) at −80 °C until nucleic acid extraction. RNA and DNA from all biological samples were isolated using the AllPrep DNA/RNA micro kit (Qiagen, cat. no. 80284) following the manufacturer´s instructions, and stored at −80 °C until use. DNA and RNA were quantified by fluorometry using the Qubit dsDNA-HS and RNA-HS assays (Thermo Scientific, Waltham, MA, cat. nos. Q32854 and Q32855, respectively) and the quality of the RNA was evaluated by electrophoresis on Agilent RNA Nano Chips (Agilent Technologies, cat. no. 5067-1511).

The extraction of both DNA and RNA from the same samples was very challenging and in many cases, it was not possible to fulfill the minimum amount or quality standards required by the genome-wide techniques applied in the present study. Thus, we could not obtain all the information from all patients and although DNA methylation and SNP genotypes are available for the same individuals, RNAseq analysis has been performed in a partially different patient cohort (Table S1).

### Genome-wide methylation analysis

DNA from 20 epithelial fractions and 20 immune fractions (separated from duodenal biopsies from 10 CD patients and 10 controls) was bisulfite converted, and bisulfite-treated DNA samples were subjected to the Illumina Infinium HumanMethylation450 microarray analysis, essentially as described previously^12^. The main particularity of this part of the study was the fact that, for the first time in our laboratory, we used only 100 ng of DNA per sample, due to the limited nucleic acid availability after cell sorting.

After Infinium bead array recommended preparation and scanning^13^, raw methylation data were imported and processed using R/Bioconductor packages, including Lumi, wateRmelon and minfi^14–16^. In particular, data quality was inspected using boxplots for the distribution of methylated and unmethylated signals, and inter-sample relationship using multidimensional scaling plots and unsupervised clustering. Probes were filtered for low quality with the pfilter function^15^, and the three samples with a detection P value > 0.05 in >5% of the sites were removed (Table S1). Additionally, known cross-reactive probes^17^ were also excluded from further analysis. The remaining dataset was background-subtracted, and normalized using intra-array beta-mixture quantile normalization^18^.

Methylation beta values were logarithmically transformed to M values before parametric statistical analyses, as recommended^19^. To define differentially methylated positions (DMPs) and differentially methylated regions (DMRs), we modelled the main variables as a categorical variable (i.e. control *vs.* disease, cell subtype) in a linear regression using the limma package, an empirical Bayesian approach^20,21^. DMPs and DMRs were selected based on a FDR-adjusted p value < 0.05. DMRs were identified with the DMRcate package using the recommended proximity-based criteria^22^. In addition, a DMR was defined by the presence of at least 2 differentially methylated CpG sites with a maximum gap of 1000 bp. Functional pathway enrichment was performed with the Enrichr web application^23^. In addition, the package coMET was used for visualisation of regional associations and co-methylation patterns^24^. Differentially variable and methylated CpG sites (DVMCs) were identified using the iEVORA algorithm with the recommended parameters of adjusted and non-adjusted p values^25^.

### Direct bisulfite sequencing

In this technique, primers are designed to be strand-specific as well as bisulfite-specific, flanking (but not involving) the CpGs of interest. Thus, both methylated and unmethylated sequences will be amplified, in contrast to methylation-specific PCR. All unmethylated cytosines will be displayed as thymines in the resulting amplicon of the sense strand, and as adenines in the amplified antisense strand. This technique requires amplification of the target region by PCR prior to sequencing for adequate sensitivity. Here, we used this technique to confirm the differential methylation of the DMRs mapping to the *TAP1* and the *HLA-B* promoter in an independent cohort of complete biopsies coming from 7 GFD-treated CD patients and 7 controls.

In particular, we used the following primers to amplify 6 CpGs per gene within each previously defined DMR after bisufite conversion: TAGGGAATAGATTGAAGGTTTTAGG (forward strand) and CAATCTAACTAAAACTAACCTACTTAAACT (reverse strand), and AAATTTTTAGTGGGATAAGAAAAT (forward strand) and CCAAAAATAAACAACTATAATAATACCTTC (reverse strand) for *TAP1* and *HLA-B*, respectively, using the PyroMark PCR Kit (Qiagen, cat. no. 978703 VF 40). Afterwards, NucleoSpin Gel and PCR Clean-up (Macherey-Nagel, Düren, Germany, cat. no. 740609.250) were used to purify the pooled amplicons of each individuals. Libraries were generated with a Nextera XT kit (Illumina, cat. no. FC-131-1024) and these were sequenced in an Illumina MiSeq system by using the Miseq Reagent kit v3 (600 cycles, 25 M reads) (Illumina, cat. no. MS-102-300).

### Genotyping

The HumanImmuno v1.0 BeadChip (Illumina Inc., San Diego, CA) was used for genotyping DNA extracted from the *lamina propria* of the same patients in which the methylome study was carried out (10 CD and 10 controls) at the CIC Biogune Genomics Platform, Derio-Bizkaia, Spain, according to Illumina protocols. SNP mapping and allele calling were done essentially as described in the original Immunochip article^1^. To search for interactions between SNPs and methylation levels we adapted the Matrix eQTL package and checked the correlations within 5 Kb upstream and downstream of each of the polymorphisms (FDR-corrected p < 0.05)^26^.

### Transcriptome analysis

Total RNAseq was performed in (a) 22 epithelial fractions of biopsies taken from 10 CD patients and 12 control individuals without intestinal inflammation at the time of endoscopy; (b) 12 immune fractions of biopsies taken from 7 celiac and 5 control individuals, and (c) 8 complete biopsies taken from 4 celiac and 4 control individuals at the CIC Biogune Genomics Platform and the Beijing Genomics Institute.

Libraries were prepared using the TruSeq Stranded Total RNA kit (Illumina Inc., cat. no. RS-122- 2201), following the TruSeq® Stranded Total RNA Sample Preparation Guide. Starting from 50 ng of total RNA, Eukaryotic rRNA was depleted and remaining RNA was purified, fragmented and primed for cDNA synthesis. cDNA first strand was synthesized with SuperScript-II Reverse Transcriptase (Life Technologies, cat. no. 18064–014) for 10 min at 25 °C, 15 min at 42 °C, 15 min at 72 °C. Second strand cDNA was synthesized with Illumina reagents at 16 °C for 1 hour. Then, A-tailing and adaptor ligation were performed. Finally, enrichment of libraries was achieved by PCR (30 sec at 98 °C; 15 cycles of 10 sec at 98 °C, 30 sec at 60 °C, 30 sec at 72 °C; 5 min at 72 °C).

On the other hand, libraries were visualized on an Agilent 2100 Bioanalyzer using Agilent High Sensitivity DNA kit (Agilent Technologies, cat. no. G2938-90320) and quantified using quantitative PCR with Kappa Library Quantification Kit (Master Mix and DNA Standards, KAPA – Biosystems cat. no. KK4824) and Qubit dsDNA HS DNA Kit (Life Technologies, cat. no. 32851). Paired-end sequencing of 75 nucleotides was carried out in a HiScanSQ platform (Illumina Inc.). Libraries were pooled and each pool sequenced in two lanes at a final concentration of 3.7 pM (each library was intended to be sequenced at 0.458 pM per lane). On average we generated 30 +/− 2.5 (mean +/− SD) million reads per sample.

Sickle was used to remove low quality reads, -n 13 option was used and the rest of options set to the default values^27^. The Tuxedo protocol was used to map sequenced reads against the human reference genome (Hg38) with Tophat and providing GENCODE 24 as reference transcriptome^28,29^. Cufflinks was used to assemble the transcriptome using de novo transcript search and to calculate the FPKM value of each transcript, to test the differences between health and disease, and to get the normalized values^30^. Additionally, DESeq2 and edgeR were used to detect differential expression related to CD (FDR < 0.02) in the form of normalized counts^31,32^. In both cases, we used the number of raw counts calculated by Cufflinks. In the case of edgeR “*quasi* likelihood” analysis was used. We considered a particular gene to be expressed when raw counts >5 in more than the 50% of the samples.

## Results

### Separation of the epithelial and the immune cellular fractions from intestinal biopsies

Taking advantage of the fact that epithelial cells express the epithelial cell adhesion molecule EpCAM (CD326) on their surface^33^ while immune cells harbor the CD45 antigen encoded by the *PTPRC* gene^34^, we developed a rapid procedure for the separation of the two cell populations from duodenal biopsy samples in order to perform independent DNA methylation studies in each of them. It is important to bear in mind the difficulty to obtain enough DNA from both cell fractions.

**Figure 1:**
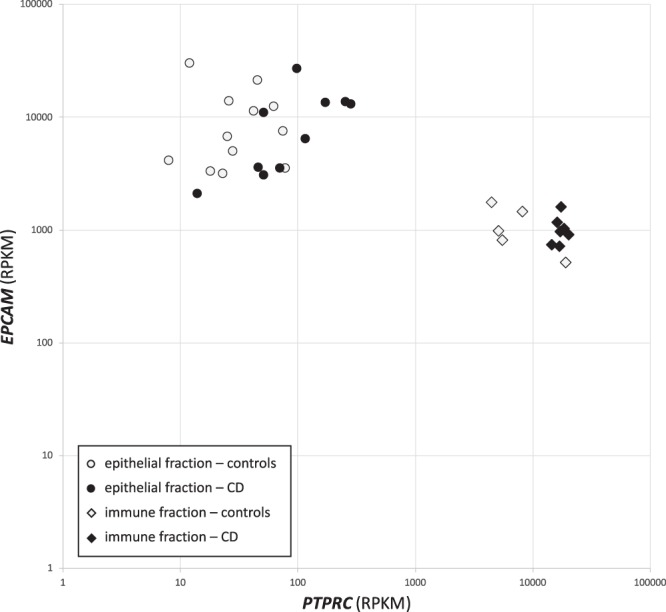
The efficiency estimation of the cell separation. The relative expression (normalized RNAseq counts) of *EPCAM* and *PTPRC* was used to account for the efficiency of the cell separation through the estimation of the proportion of the epithelial cells and the IELs, respectively. The purity of the epithelial and the immune fraction (expressed as *EPCAM*:*PTPRC* and *PTPRC*:*EPCAM*) was 99 ± 1% and 90 ± 7%, respectively.

In this context, the relative expression of *EPCAM* and *PTPRC* was used to account for the efficiency of the cell separation through the estimation of the proportion of the epithelial cells and the intraepithelial lymphocytes (IELs), respectively. More specifically, we took into consideration the normalized counts of these two genes in our RNAseq study. The purity of the epithelial fraction (expressed as *EPCAM*:*PTPRC*) was 99 ± 1% (Fig. 1), while the average purity of the immune fraction (*PTPRC*:*EPCAM* ratio) was 90 ± 7%. It is important to stress that there were no significant differences in the enrichment for the immune cellular fraction between control and CD samples after separation, reducing the DNA methylation bias that the different IEL proportion in whole tissues would generate.

### Differentially methylated positions in epithelial and immune cell fractions of the celiac duodenal mucosa

To investigate the methylation profile in CD, we analyzed 40 DNA methylomes from 10 control individuals without intestinal inflammation when endoscopy was performed and 10 CD patients at diagnosis. DNA for methylation analyses was extracted from both the epithelial and the immune cell compartments from all the individuals in the study. The association tests for disease status in the form of a Q-Q plot (Fig. S1) showed a lambda factor of 1.2 and therefore, a minimal inflation of the results.

**Figure 2:**
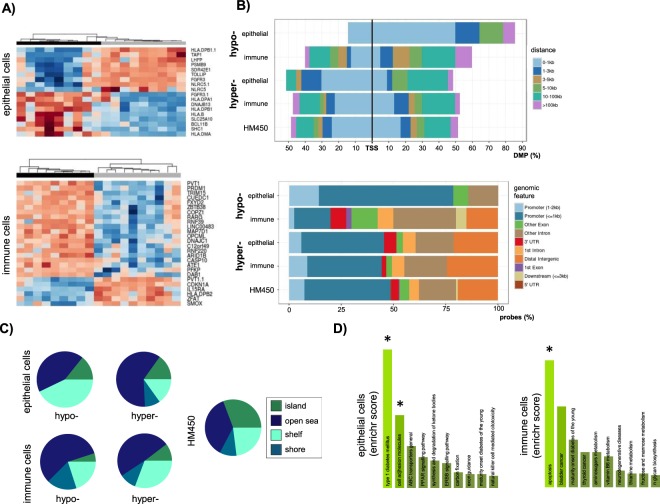
General DMP analysis for CD status in each cell type. (**A**) Heatmap showing active CD (black) and controls (grey) in columns and the genes closest to the most differentially methylated DMPs in rows, with the blue-red color scale representing the hypo- to hypermethylation trend. (**B**,**C**) Distribution of DMPs, relative to transcription start sites, genomic features and CpG content and characterization, respectively. (**D**) KEGG pathway analysis of the DMPs. All analyses were performed with FDR < 0.05 and delta-beta > 10% cuttoffs, except for the heatmap (delta-beta > 20%).

Comparison between CD patients and controls identified 43 differentially methylated positions (DMPs) (11 hypo and 32 hypermethylated) and 310 DMPs (40 hypo and 270 hypermethylated) in the epithelial and immune fractions, respectively, using FDR <0.05 and delta-beta >10% as thresholds (Table S2). In the epithelial compartment, 18 hits with delta-beta >20% and located in close proximity to 15 different genes, were able to perfectly discriminate CD and control samples (Fig. 2A). We also observed that the hypomethylated positions seemed to be more abundant in promoter and transcription start site (TSS) proximal regions, with a slight decrease of CpG islands (CGI) (Fig. 2B,C), but these results were not significant. The most represented pathways among the differentially methylated hits were *type 1 diabetes* and *adhesion molecules* (FDR < 0.05) (Fig. 2D), coherent with our prior knowledge on the disease.

On the other hand, in the immune compartment, 26 hits with delta-beta >20% and located close to 25 different genes, were able to unambiguously cluster CD and control samples (Fig. 2A). The analysis of the immune cell DMP set showed a significant decrease of CGI (χ^2^ > 7.950, p = 0.0048 and χ^2^ > 31.489, p < 0.00001 for hypo and hypermethylated DMPs, respectively) together with an enrichment of open sea regions (χ^2^ > 3.411, p = 0.0648 and χ^2^ > 8.948, p = 0.0028 for hypo and hypermethylated DMPs, respectively) (Fig. 2B,C). Interestingly, *apoptosis* was the main enriched pathway in the genes close to the immune cell-specific DMPs (FDR < 0.05) (Fig. 2D).

RNAseq results from epithelial fractions from 10 CD patients and 12 controls, and immune fractions from 7 patients and 5 controls (Table S3) showed that 11/15 and all 25 differentially methylated genes were expressed in the epithelial and immune cell compartments, respectively. Seven of the eleven genes expressed in the epithelial fraction (*HLA-DPB1*, *TAP1*, *PSMB9*, *HLA-DPA1* and *HLA-B* in the HLA region on chromosome 6, and *SDR42E1* and *NLRC5*, located elsewhere) were differentially expressed in CD (Fig. S2). Four out of those seven genes (*TAP1* and *PSMB9* in the HLA, and *SDR42E1* and *NLRC5*) were also differentially expressed in the immune fraction. In contrast, out of the 25 genes close to the immune DMPs only *IL15RA* and *ZFAT* were overexpressed, in both cell populations.

### Differentially variable and methylated CpG sites in epithelial and immune cell fractions of the celiac duodenal mucosa

**Figure 3:**
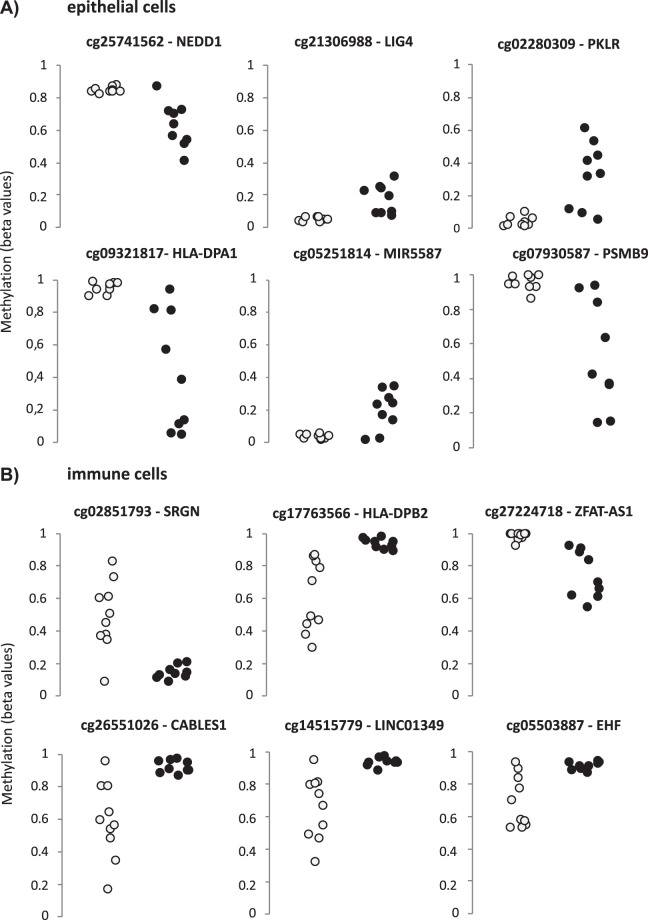
Most variably and differentially methylated positions in each cell type. The figure shows the top DVMCs per cell fraction, according to the iEVORA R package, as described in *Methods*. Grey and black circles represent control and celiac samples, respectively.

Increased variation in DNA methylation has been proposed as a characteristic of certain conditions, including premalignant stages^35^. We identified 560 differentially variable and differentially methylated CpGs (DVMCs) associated to CD in the epithelial cell population, out of which 93.6% were more variable in the disease group, and were borderline significant for the *IBD pathway* (FDR = 0.05798). In turn, there were 851 celiac-associated DVMCs in the immune cells, but only 26% were more variable in CD, and were not enriched in any particular term (Table S4, Fig. 3). According to the RNAseq results, four of the genes that were close to the six most significant DVMCs of the epithelial fraction were differentially expressed in CD; namely *NEDD1*, *PSMB9*, *PKLR* and *HLA-DPA1*. In general, DVMCs seemed to be related to fewer changes in gene expression in the case of the immune fraction (Table S3, Fig. S3).

### Cell population-specific differentially methylated regions in celiac disease

Since DNA methylation of neighbouring CpG sites can be highly correlated, we applied dimension reduction to search for CpG clusters that define differentially methylated regions (DMRs) (Table S5). We found six DMRs in epithelial cells, while 100 were significant in the immune cell compartment (only significant DMPs were taken into consideration for DMR identification). Taking into account the strong linkage disequilibrium in the HLA region, we classified DMRs into those on that region of chromosome 6 and those located elsewhere in the genome. Four epithelial and 7 immune DMRs were located in the HLA region, and two of them (*TAP1* and *TRIM15*) were common to both cell populations. The limited overlap between the two cell populations supports the idea that disease-related differences in DNA methylation present only in one of the cell types, could be genotype-independent, cell type-specific epigenetic marks that respond to the environmental component of the disease.

**Figure 4:**
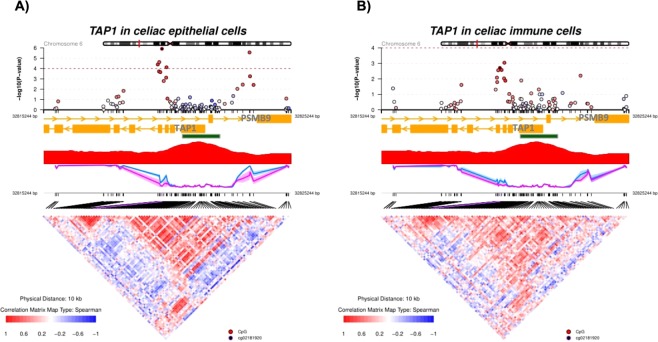
Methylation and comethylation profiles of the HLA-DMR close to *TAP1*, in celiac epithelial (**A**) and immune (**B**) cells. The graphs at the top show the p values of the methylation differences between celiac and control cells in each CpG position. The black circle is the reference hit, and the color of the rest of the circles represents the Spearman correlation coefficient between the rest of the methylation values and the reference point, according to the color scale. The CG content is shown in red and the blue and the purple lines below represent the methylation levels in the control and the celiac patients, respectively. The squares in the correlogram at the bottom represent the pairwise correlation between CpG positions.

One of the most significant HLA-DMRs in the epithelial compartment mapped to *TAP1*, and consisted of two small regions surrounding a CGI in its gene promoter that were significantly hypomethylated in epithelial cells from CD patients, and showed a weaker trend in immune cells (Fig. 4). The hypomethylated region also overlapped with an intron of *PSMB9*. Additionally, comethylation was much stronger in epithelial cells compared to their immune counterparts, suggesting a higher degree of coordination in the former. In the case of *HLA-B*, with a DMR exclusive for the epithelial fraction, we also found a significantly hypomethylated CGI shore in its promoter region (Fig. S4). Interestingly, the XXbac-BPG248L24.12 pseudogene overlapped the most CpG-dense *locus* of the surroundings. On the other hand, the top non-HLA DMRs in celiac disease mapped to genes related to the immune response, including *NLRC5* and *TMEM105* in the epithelial fraction, and *CAST* and *HOXC4* in the immune compartment (Fig. S5).

From a structural point of view, the most significant DMRs exhibited loss of CGI boundaries, or the blurring of the demarcated borders between high and low methylated regions defined by CGIs, a feature that has been described in cancer^36^. In general, boundary shifts into CGIs appear as hypermethylated islands while boundary shifts outside the islands appear as hypomethylated shores^37,38^. The former is observed in *CAST* and *HOXC4* and, to a lesser extent, in *TMEM105*, since significant hypermethylation is found in the most CpG-dense region of the surrounding genomic landscape (10 Kb). On the contrary, CGI shores are hypomethylated in the rest of the top DMRs, particularly *HLA-B*, *NLRC5* and *TAP1*.

### Validation of the differential methylation of the *TAP1* and the *HLA-B* promoters in the HLA region in an independent cohort of treated celiac patients

**Figure 5:**
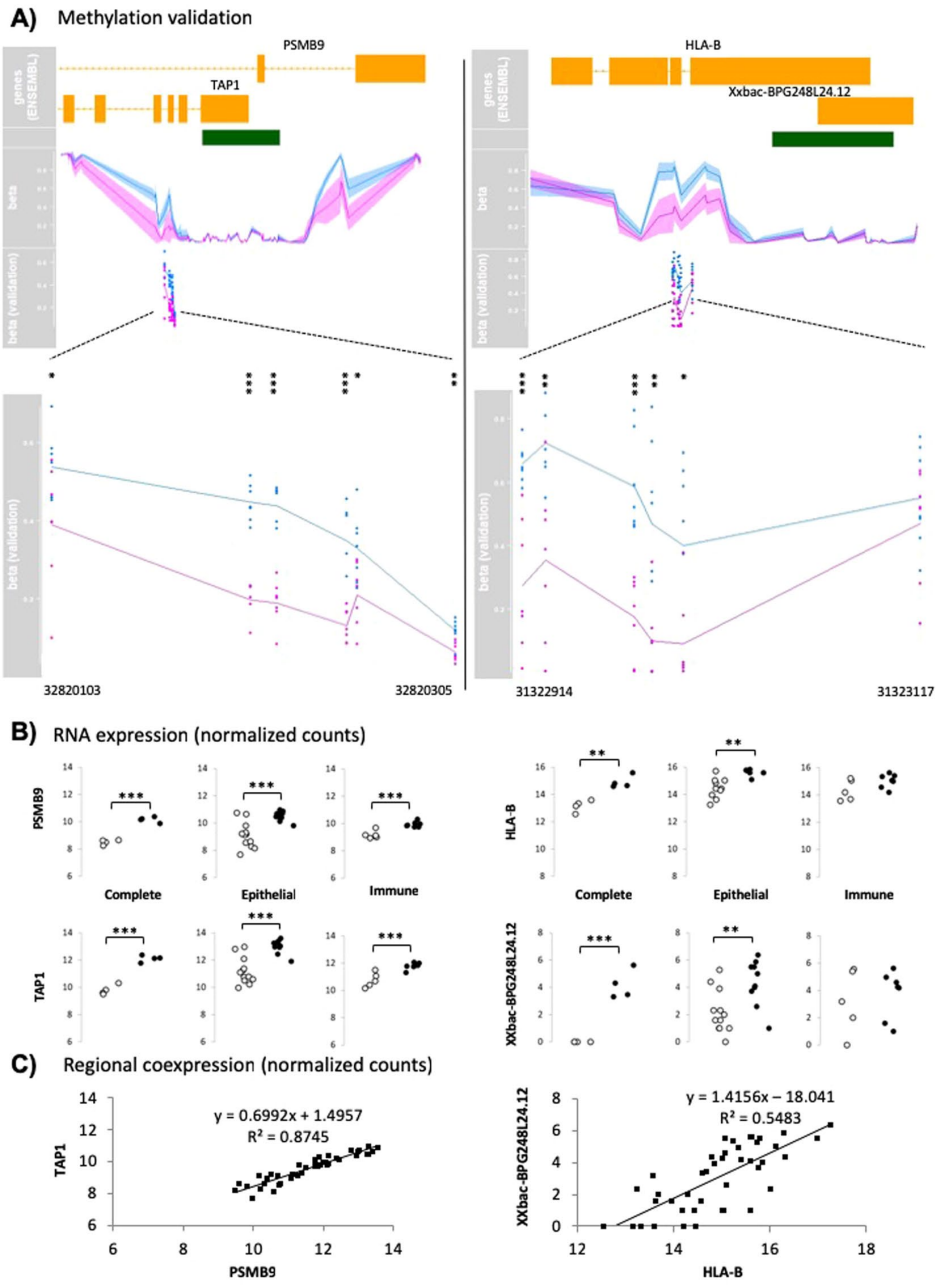
Validation of the *TAP1* and *HLA-B* overlapping DMRs by direct bisulfite sequencing and expression analysis. (**A**) Genomic context and methylation patterns of CD patients (purple) and control individuals (blue) in each DMR. Methylation levels extracted from the methylation array are represented in the upper panel, while those obtained after direct bisulfite sequencing appear below and are referred to as “betas (validation)”. Green boxes represent CGIs. *, ** and *** represent p values < 0.05, 0.01 and 0.001 (unpaired T-test), respectively. (**B**) Expression patterns of the DMR-overlapping genes in the different cell compartments. Grey and black dots represent control and CD patients, respectively. (**C**) Coexpression of the regional gene-pairs.

Contrary to the reccomendations in force several years ago, current diagnosis protocols for CD do not include a control biopsy of patients on a gluten-free diet (GFD). Samples from asymptomatic, GFD-treated patients are very valuable given that genomic alterations are likely to reflect constitutive characteristics related to their genetic or epigenetic predisposition, that have been either inherited or acquired very early in childhood (before CD onset) or even in prenatal stages. In this context, DNA methylation was analyzed by NGS in bisulfite-treated, PCR-amplified fragments spanning the *TAP1* and *HLA-B* DMRs in an independent cohort of GFD-treated CD patients and control individuals. Both DMRs are differentially methylated in DNA from complete biopsies of asymptomatic CD patients. In particular, we studied 6 CpG sites per *locus* and the differences between GFD-treated CD patients and controls were very significant for most of them (Fig. 5A).

According to our RNAseq results, *TAP1* and *PSMB9* were overexpressed in disease in all sample types (Fig. 5B) while the upregulation of *HLA-B* (and its overlapping pseudogene) was observed only in epithelial cells and in complete biopsies. These results are consistent with the fact that the *HLA-B* DMR is specific to the epithelial population. Additionally, both the *TAP1-PSMB9* and the *HLA-B*-pseudogene pairs were regionally coexpressed across the entire dataset (Fig. 5C), suggesting a common regulatory mechanism.

### Methylation quantitative trait *loci* and genotype-methylation interaction analyses in duodenal cell fractions

In order to ascertain whether methylation changes overlap sequence variants potentially explaining the phenotypic effect of some of the previously associated SNPs, methylation results were analyzed together with the Immunochip SNP genotypes^39^. We identified 1,423 and 1,457 mQTLs in epithelial and immune cells, respectively (FDR < 0.05), using previously defined criteria^40^ (Table S6). The mQTL-SNPs were more consistent between cell types than the CpGs they regulated (Fig. S6). In fact, 46.5% of the SNPs participating to epithelial-mQTLs also took part in immune-mQTLs, while only 30.7% of the CpGs in epithelial-mQTLs were also present in the immune (Monte Carlo-corrected χ2 = 75.036, p < 9.99 × 1e-5). Additionally, there was an excess of promoter and TSS proximal regions in both epithelial and immune mQTL-SNPs compared to a random list of Immunochip SNPs (T-test, p value < 0.001). The mQTL-SNPs identified in each particular cell type were enriched in enhancer elements that have been described in cell lines resembling each of the two cellular compartments, according to the Dragon ENhancers database (DENdb)^41^. Particularly, 21.7% of the SNPs participating to the epithelia-specific mQTLs were located in epithelia-specific enhancers, compared to the 13.3% that mapped to non-specific enhancers; and 32.1% of the SNPs participating to the immune fraction-specific mQTLs were located in immune-enhancers, compared to the 12.4% that mapped to the non-specific ones (Monte Carlo-corrected χ2 = 52.114 and χ2 = 88.197, p < 9.99 × 1e-5, respectively).

**Figure 6:**
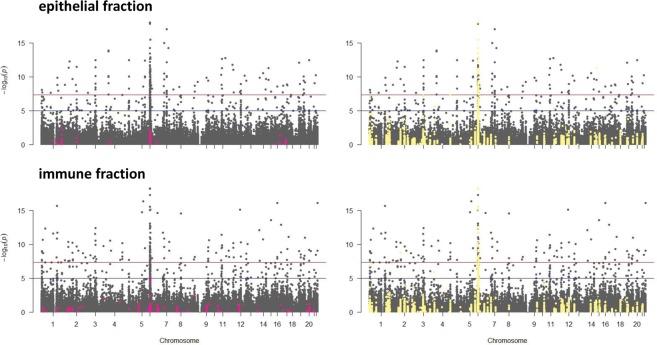
mQTLs and their overlap with differential methylation and genetic association in CD. Manhattan plot of the p values of the putative *cis-*mQTLs identified in epithelial (top) and immune (bottom) cells. mQTL-SNPs that correlated with individual DMPs are shown in pink (none of the DMP-correlated SNPs reached genome-wide significance in the putative *cis*-mQTLs in which they participated). In turn, SNPs associated to CD according to the Immunochip project^1,39^ are highlighted in yellow, and consequently, mark those mQTLs that overlap CD-associated variants, many of which are genome-wide significant.

In general, methylation levels of the CpGs regulated by the mQTLs did not differ between celiac patients and controls (Fig. 6), suggesting that the celiac methylation signature described above is independent from the mQTL-SNP genotypes. The only exception was the immune cell-specific DMP in the gene body of the *HLA-DPB2* pseudogene in the HLA region, that correlated with several SNPs (Fig. S7). Although there was a remarkable effect of the genotype in both the epithelial and immune cell fractions, the contribution to disease was restricted to the immune population, further supporting the idea of additional disease-predisposing factors other than genotype in the HLA region.

In order to asses the proportion of associated SNPs that are cell type-specific mQTLs, we searched for overlaps between the mQTL-SNPs and the most CD-associated SNPs (n = 7,144; p < 1e-4) from the Immunochip compared to the less associated 7,144 variants. We found that 363/1423 and 311/1457 overlapped, resulting in very significant enrichments for both epithelial (Monte Carlo random sampling-corrected χ2 = 226.97, p = 9.99 × 1e-05) and immune (Monte Carlo random sampling-corrected χ2 = 182.14, p = 9.99 × 1e-05) compartments, respectively (Fig. 6). As expected, most of them mapped to the HLA region on chromosome 6 but there were some that mapped to other chromosomes, such as 15 CD-associated SNPs spanning 471,524 bp in the chemokine receptor cluster on chromosome 3 that were correlated to the methylation level of four different CpG sites in the epithelial cell fraction. Interestingly, 178 CD-associated SNPs participated in mQTLs with regulatory ability in the two cellular fractions, while the rest were cell type-specific. However, when those mQTLs mapping to the HLA region were excluded, only 13 SNPs that correlated with the methylation level of a single position (cg21209485) and were located in the gene body of *MMEL1* on chromosome 1, appeared to be common to both cell types (Table S7).

Finally, we wanted to ascertain whether there is an enrichment of DMPs around CD-associated variants. For this purpose, we questioned whether CD-associated DMPs colocalize with CD-associated Immunochip *loci*^18^ and therefore could be at least partially explained by sequence variation. We could not find any epithelial nor immune cell-specific DMP within defined distances of CD-associated sites (bin sizes of 25, 50, 100 and 250 Kb around each SNP). In turn, we found increasing numbers of DMPs in the bins of our randomly picked Immunochip/genome-wide SNP sets of the same size, also after Monte Carlo sampling correction, probably due to the broader representation of the genome. Moreover, when we took CD-associated regions as a whole instead of single positions, we found little, non-significant overlap with DMPs when compared to similar, non-associated regions of the Immunochip, consisting on two isolated CpGs in chromosomes 2 and 15 that were differentially methylated in the immune fraction.

## Discussion

Here we present the first methylome study performed in duodenal mucosa samples from CD patients. We describe two profoundly different cell-type specific methylation profiles that are both characterized by a remarkable loss of CGIs. However, the DMP overlap between the epithelial and immune compartments is limited and restricted to the HLA region, given that two out of the 4 epithelial cell-specific DMRs in that region are also detected in the immune compartment. It is hard to tell whether the signal in immune cells is real or a consequence of the small (<10%) existing epithelial cell contamination of the immune fraction combined with a very pronounced differential methylation in the epithelium. Overall, the minimal overlap of differentially methylated hits between the two different cell populations and the negligible inflation of the raw methylation results suggest that the limited sample size can be circumvented, at least in part, by the cell separation strategy, in terms of the robustness of the results.

On the other hand, expression analysis of the genes located in close proximity to the cell type-specific DMPs showed that in general, methylation alterations in the epithelial cell compartment have a greater functional impact compared to those in the the immune fraction. Interestingly, many of the HLA genes affected by these methylation changes in the celiac epithelium were not only expressed in our dataset but also differentially expressed depending on the disease status. Nevertheless, it is important to note that even if expression alterations in genes mapping close to the immune-specific DMPs were limited, they affected interesting genes such as *IL15RA* and *ZFAT*, that appeared to be overexpressed. The former is a well-known mediator of IL15 activity that is associated to CD^42^ and that can promote IEL survival in the disease, leading to the emergence of T cell clonal expansion^43^. In turn, *ZFAT* was originally identified as a candidate susceptibility gene for autoimmune thyroid disease and its deficiency in peripheral T cells results in a reduction in T cell counts with decreased expression of several interleukins^44^.

Regarding differential variation of methylation levels between disease groups, the results in the epithelial fraction are in line with previous findings that point to chronic inflammatory and precancerous stages as more prone to methylation variation^35^. In fact, the top two most significant DVMCs in epithelial cells mapped to an intergenic region several kilobases upstream of the promoter of *NEDD1*, a gene that has been related to gastric cancer^45^ and to a TSS-proximal intron in *LIG4*, a gene that harbors a recently discovered pathogenic variant responsible for IBD development^46^. In general, we found a borderline enrichment of IBD-related terms among the genes mapping close to the epithelial DVMCs. Additionally, the disease-associated changes in expression of the genes close to the top hits further support the idea of functionality of the methylation alterations observed. In contrast, the immune fraction shows the opposite trend when compared to the epithelial compartment. A plausible explanation to the lower variation in the immue compartment of CD patients could be that there is a small number of expanded clones dominating the T-cell repertoire in the disease^47^ and thus reducing the heterogeneity of the immune cell population. Moreover, genes close to immune DVMCs were not enriched in any particular term and were generally not differentially expressed in CD, reinforcing the idea that loss of variation is more a consequence of clonal expansion rather than a functional feature. Overall, our results suggest that this approach could be an interesting tool for new target discovery in inflammatory conditions like CD.

The fact that most of the top DMRs identified both in and outside the HLA region exhibited loss of CGI boundaries, together with the finding that methylation levels of celiac epithelial cells are more variable than the same *loci* in controls, lead to the idea of celiac intestinal epithelia evoking precancerous stages of solid tumours, where there is a well described loss of boundaries between high and low methylation regions defined by CGIs^36^. Remarkably, several of the DMRs located outside the HLA region target genes related to the immune response, like *NLRC5* and *CAST*. The former is an MHC class I regulator usually expressed in epithelial cells that has been shown to protect T lymphocytes from NK-cells in inflammatory conditions, and CAST plays a central role in the NFkB-mediated regulation of macrophage activation in inflammatory disorders of the gut^48,49^. Interestingly, *NLRC5* shows a very significant hypomethylation of its CpG-dense promoter and it is overexpressed in CD.

An important result of this work is the observation of (at least partially) genotype-independent methylation changes in the HLA region, as shown by the fact that they are associated with the disease in a cell population-specific manner and are not under the control of any Immunochip-SNP in our mQTL analysis. Although a study on chronic HIV infection points towards differential methylation as a dynamic and progressive mechanism that can alter the expression patterns of HLA genes^50^, the majority of reports so far do not discard the effect of the strong linkage disequilibrium in the region or even describe those changes as fully haplotype-dependent^51^. However, more recent works in the field have provided evidence supporting methylation as an independent layer of variation with functional effects in this complex genomic region, as can also be inferred from our results. This is the case of an interesting study on high-risk human papillomavirus, whose infection is able to repress HLA-E expression through methylation alteration^52^. Additionally, hypermethylation of the MHC locus *RNF39* has been found to be associated with multiple sclerosis, accounting for a portion of risk independent from the well-stablished susceptibility conferred by *HLA-DRB1*^53^.

Moreover, we were able to confirm the differential methylation of two different *loci* within the HLA region in an independent collection of complete biopsies coming from GFD-treated patients, particularly the *TAP1* and *HLA-B* promoters. Very interestingly, expression results seem to be coherent with the cell type-specificity of the differential methylation around *HLA-B*. Altogether, these results are suggestive of a non-reversible epigenetic celiac signature that prevails after more than two years on GFD. Alternatively, these results could also suggest that certain methylation alterations are independent of gluten exposure and were acquired very early in life or even during fetal development. We propose these two elements and their overlapping genes as novel and independent candidates in the region. Particularly, *TAP1* is an important HLA class-I surface peptide, and recent works have shown that impairement of this epigenetically regulated gene is responsible of the escape of cancer cells from the host’s immuno-surveillance^54,55^. In turn, the exacerbated expression of *TAP1* observed in CD could indicate an anomalous boost of the adaptive immune system.

Finally, mQTL analysis revealed that at least a part of the celiac methylation signature is independent from the genotype. Since only Immunochip SNPs in *cis* have been used to build up the mQTL SNP-CpG pairs, we cannot rule out that a number of differentially methylated hits are regulated by non-genotyped SNPs within the Immunochip regions or in *trans* with other variants elsewhere in the genome, although the frequency of these two cases is expected to be minimal. These findings oppose recent methylation and mQTL analyses in IBD by Ventham *et al*. in which they conclude that differential methylation is driven by genetic variation^56^. However, our results are still compatible with this idea given that first, the immunogenic insult and thus, the environmental factors driving disease are completely different in CD and IBD. Also, our sample size is more limited and consequently we are probably not able to see the genetic variation-dependent methylation changes, due to the small differences in allele frequencies between patients and controls. In fact, there is a remarkable enrichment of SNPs participating to mQTLs in CD-associated variants that nevertheless regulate CpG positions that do not differ in disease. This was the case of the SNPs located in the gene body of *MMEL1* on chromosome 1, that regulated a single CpG position in both cell populations, or of the variants spanning a 0.5 Mb region on chromosome 3 that harbors several chemokine receptor genes, including *CCR5*, whose methylation levels have previously been associated to its expression^57^.

In conclusion, in CD, the two main cell populations of the duodenal mucosa present divergent methylation signatures that in the case of the epithelial fraction is reminiscent of precancerous solid tumors, harboring a greater variability and affecting several *loci* in the HLA region in a genotype-independent manner. Additionally, the association of several SNPs with CD could be explained by allele-specific methylation such as the case of the SNPs located in the *MMEL1 locus*. Finally, we can conclude that our results confirm previous studies^58^, according to which genome sequence variation has a broad effect on phenotype across cellular compartments, whereas epigenetic factors provide an additional layer of variation that is more tissue or even cell type-specific.

## Supplementary information

Supplementary Information

Supplementary Table S2

Supplementary Table S3

Supplementary Table S4

Supplementary Table S5

Supplementary Table S6

Supplementary Table S7

## Data Availability

Methylation and SNP genotype results are accessible in NCBI’s Gene Expression Omnibus (GEO) repository through accession no. GSE84745 (https://www.ncbi.nlm.nih.gov/geo/query/acc.cgi?acc=GSE84745), and RNAseq data have been deposited in NCBI’s Sequence Read Archive (SRA) with accession no. SRP077708 (http://www.ncbi.nlm.nih.gov/sra/SRP077708).
