# Supplementary material for: The methylome of the celiac intestinal epithelium harbours genotype-independent alterations in the HLA region
Source: Sci Rep. 2019 Feb 4;9:1298. doi: 10.1038/s41598-018-37746-6 (PMC6362130; doi:10.1038/s41598-018-37746-6)
Supplement: Supplementary file 1 — Supplementary Information [file 41598_2018_37746_MOESM1_ESM.pdf]

SUPPLEMENTARY INFORMATION

The methylome of the celiac intestinal epithelium harbours genotype-independent alterations in the HLA region

Nora Fernandez-Jimenez, Koldo Garcia-Etxebarria, Leticia Plaza-Izurieta, Irati Romero-Garmendia, Amaia Jauregi-Miguel, Maria Legarda, Szilvia Ecsedi, Ainara Castellanos-Rubio, Vincent Cahais, Cyrille Cuenin, Davide Degli Esposti, Iñaki Irastorza, Hector Hernandez-Vargas, Zdenko Herceg, Jose Ramon Bilbao

Supplementary Figure Legends

S1 Fig. Q-Q plot of the raw methylation results.

S2 Fig. Expression of the closest genes of the top epithelial-DMPs. Grey and black dots represent controls and CD patients at diagnosis (CD), respectively. *, ** and *** represent p values < 0.05, 0.01 and 0.001, respectively, for the comparisons performed in the epithelial fraction. On the other hand, $^{+}$, $^{++}$ and $^{+++}$ represent p values < 0.05, 0.01 and 0.001, respectively, for the comparisons carried out for the same genes in the immune compartment, although data-points are not shown. All comparisons were performed by unpaired T-tests.

S3 Fig. Expression of the closest genes of the top epithelial (A) and immune (B) DVMCs. Grey and black dots represent controls (C) and CD patients at diagnosis (D), respectively. *, ** and *** represent p values < 0.05, 0.01 and 0.001, respectively. On the other hand, $^{+}$, $^{++}$ and $^{+++}$ represent p values < 0.05, 0.01 and 0.001, respectively, for the comparisons carried out for the same genes in the opposite compartment (where discovery was not carried out). All comparisons were performed by unpaired T-tests.

S4 Fig. HLA-B, one of the top HLA-DMRs exclusive of the celiac epithelial compartment in the epithelial (a) and the immune (b) fraction. Circles represent the p values of the methylation difference between celiac and control groups per each *locus*. The black circle is the reference hit, and the color of the rest of the circles represents the Spearman correlation coefficient among the rest of the methylation values and the reference point, as shown by the color scale. The squares in the correlogram represent the pairwise correlation among CpG positions. CG content is shown in red whereas the blue and the purple lines represent the methylation level of the control and the celiac patients, respectively.

S5 Fig. Top non-HLA DMRs. Circles represent the p values of the methylation difference between celiac and control groups per each *locus*. The black circle is the reference hit, and the color of the rest of the circles represents the Spearman correlation coefficient among the rest of

the methylation values and the reference point, as shown by the color scale. The squares in the correlogram represent the pairwise correlation among CpG positions. CG content is shown in red whereas the blue and the purple lines represent the methylation level of the control and the celiac patients, respectively.

S6 Fig. General characterization of the mQTLs. A) Venn diagrams showing CpGs (upper panel) and SNPs (lower panel) participating to mQTLs specific to each of the cell subpopulations studied (E and I meaning epithelial and immune, respectively). B) Distribution of mQTL-SNPs (%) across different genomic features and distance clusters to the nearest TSS. C) Location of mQTL-SNPs to enhancer and non-enhancer regions. e, i, r and c are abbreviators for epithelial, immune, random and common (or shared between cell subpopulations) mQTL-SNPs, respectively. Capital E represents epithelium-specific enhancers, coming from publicly available data from Hmec (mammary normal), Helas3 (epithelial carcinoma) and Nhek (normal keratinocyte) enhancers. Capital I represents immune cell-specific enhancers, coming from publicly available data from Dnd41 (T cell leukemia) and GM12878 (lymphoblastoid) enhancers. Capital R represents a random list of enhancers of the same size of the target group of each comparison. All comparisons gave significant differences among groups according to $\chi 2$ test corrected with Monte Carlo random-sampling.

S7 Fig. mQTLs and their overlap with celiac differential methylation. A) Manhattan plot of the p values of the mQTLs identified in epithelial (top) and immune (bottom) cells. DMPs are shown in green, and the only significant hit is highlighted by a red circle. B) The only mQTL overlapping a significant immune-specific DMP in the HLA-DPB2 gene body in the HLA region on chromosome 6. The grey and the green boxes show the genomic location of the SNPs participating to the mQTL and the presence of a CpG island, respectively. C) Methylation percentage of the only DMP-mQTL in epithelial (E) and immune (I) samples, according to their genotype and disease status. C: controls (grey), D: CD patients at diagnosis (black). All comparisons were performed by unpaired T-tests.

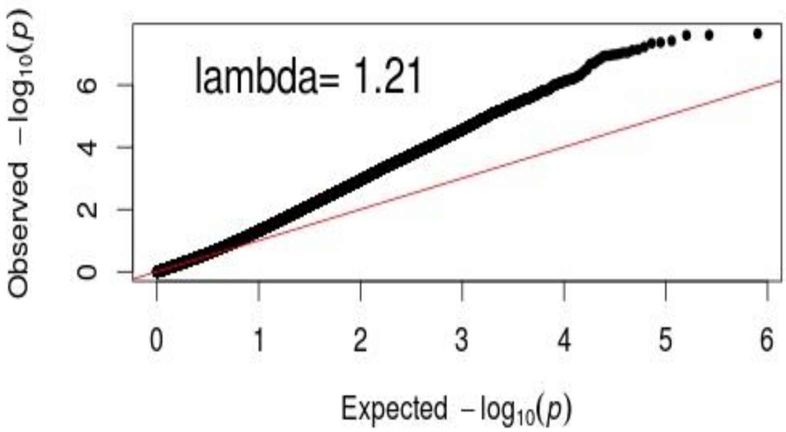
lambda= 1.21
Observed −log10(p)
Expected −log10(p)
0
2
4
6
0
1
2
3
4
5
6

S2 Fig

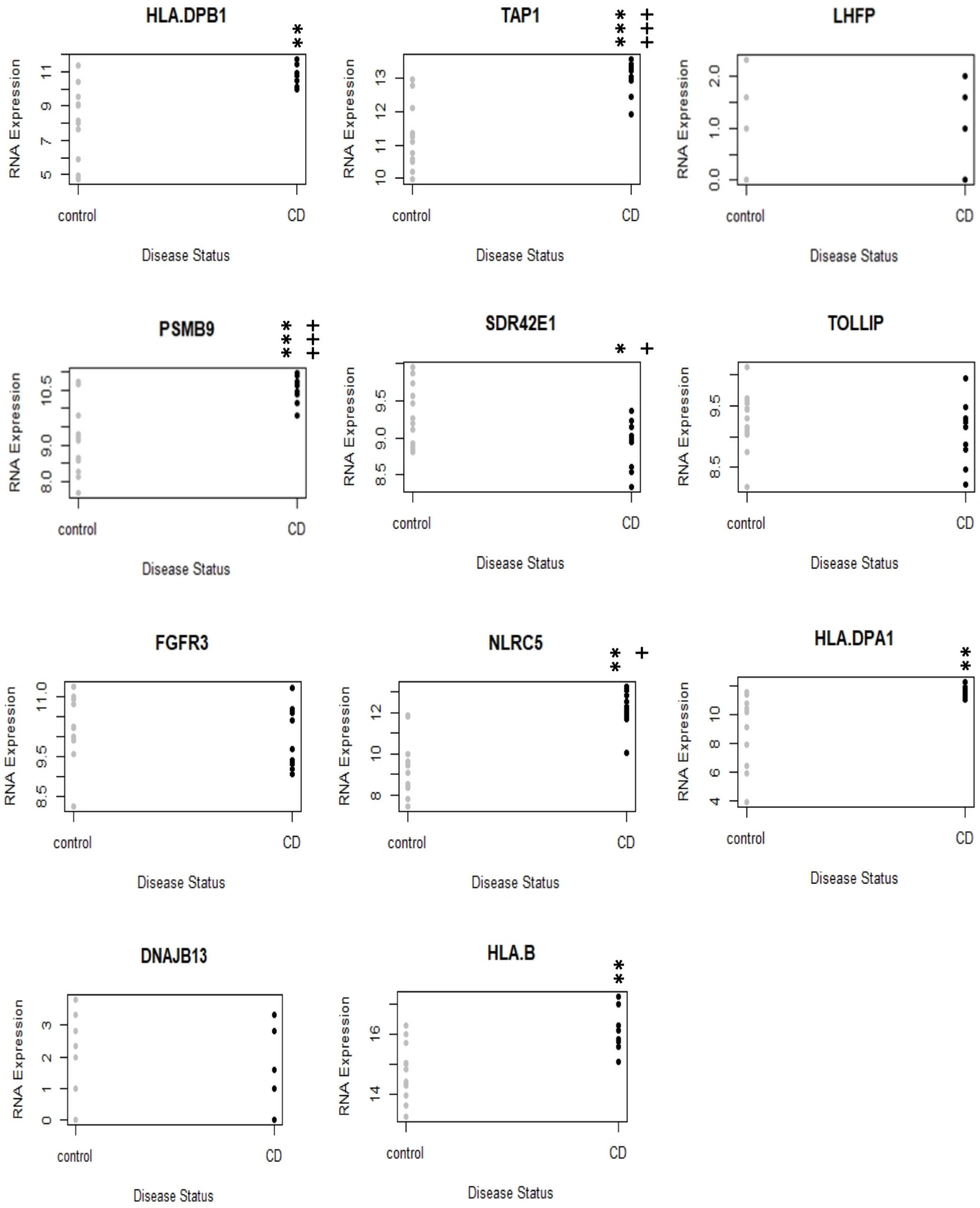
HLA.DPB1
**
RNA Expression
5
7
9
11
control
CD
Disease Status
TAP1
*** +++
RNA Expression
10
11
12
13
control
CD
Disease Status
LHFP
RNA Expression
0.0
1.0
2.0
control
CD
Disease Status
PSMB9
*** +++
RNA Expression
8.0
9.0
10.5
control
CD
Disease Status
SDR42E1
* +
RNA Expression
8.5
9.0
9.5
control
CD
Disease Status
TOLLIP
RNA Expression
8.5
9.5
control
CD
Disease Status
FGFR3
RNA Expression
8.5
9.5
11.0
control
CD
Disease Status
NLRC5
** +
RNA Expression
8
10
12
control
CD
Disease Status
HLA.DPA1
**
RNA Expression
4
6
8
10
control
CD
Disease Status
DNAJB13
RNA Expression
0
1
2
3
control
CD
Disease Status
HLA.B
**
RNA Expression
14
16
control
CD
Disease Status

A) Epithelial fraction

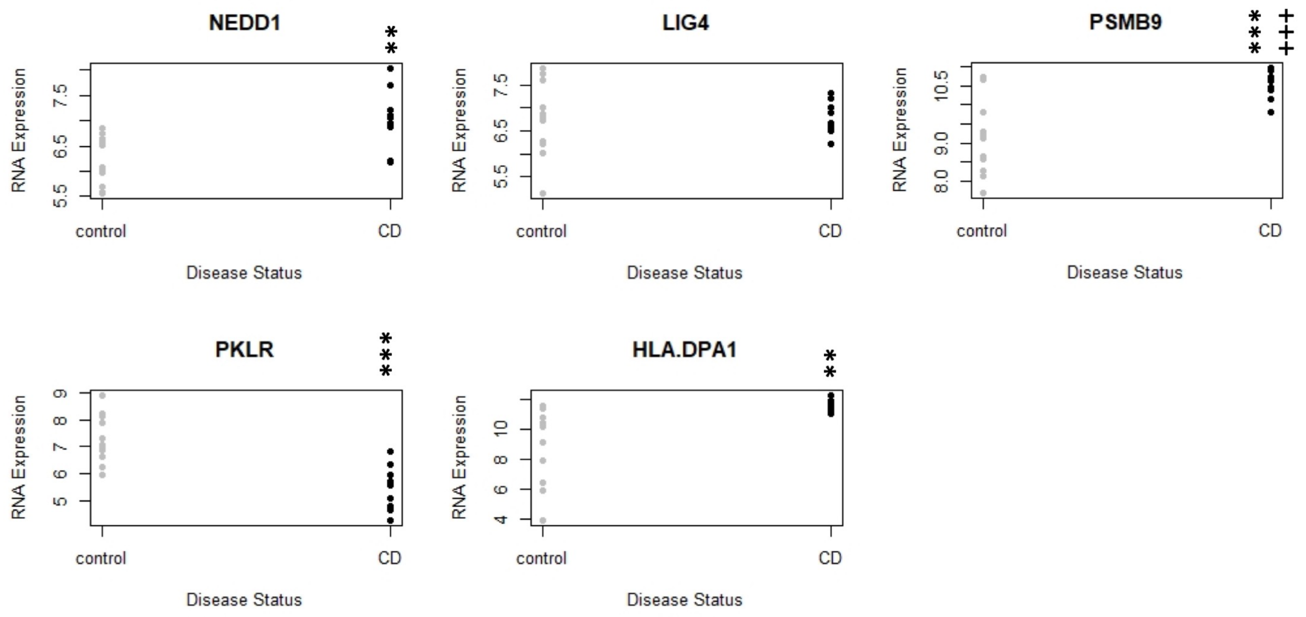

B) Immune fraction

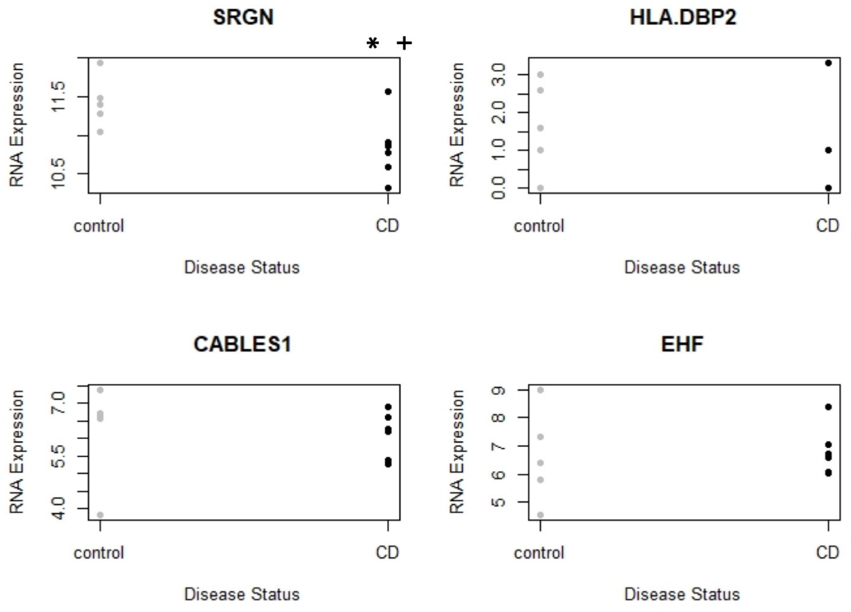

S4 Fig

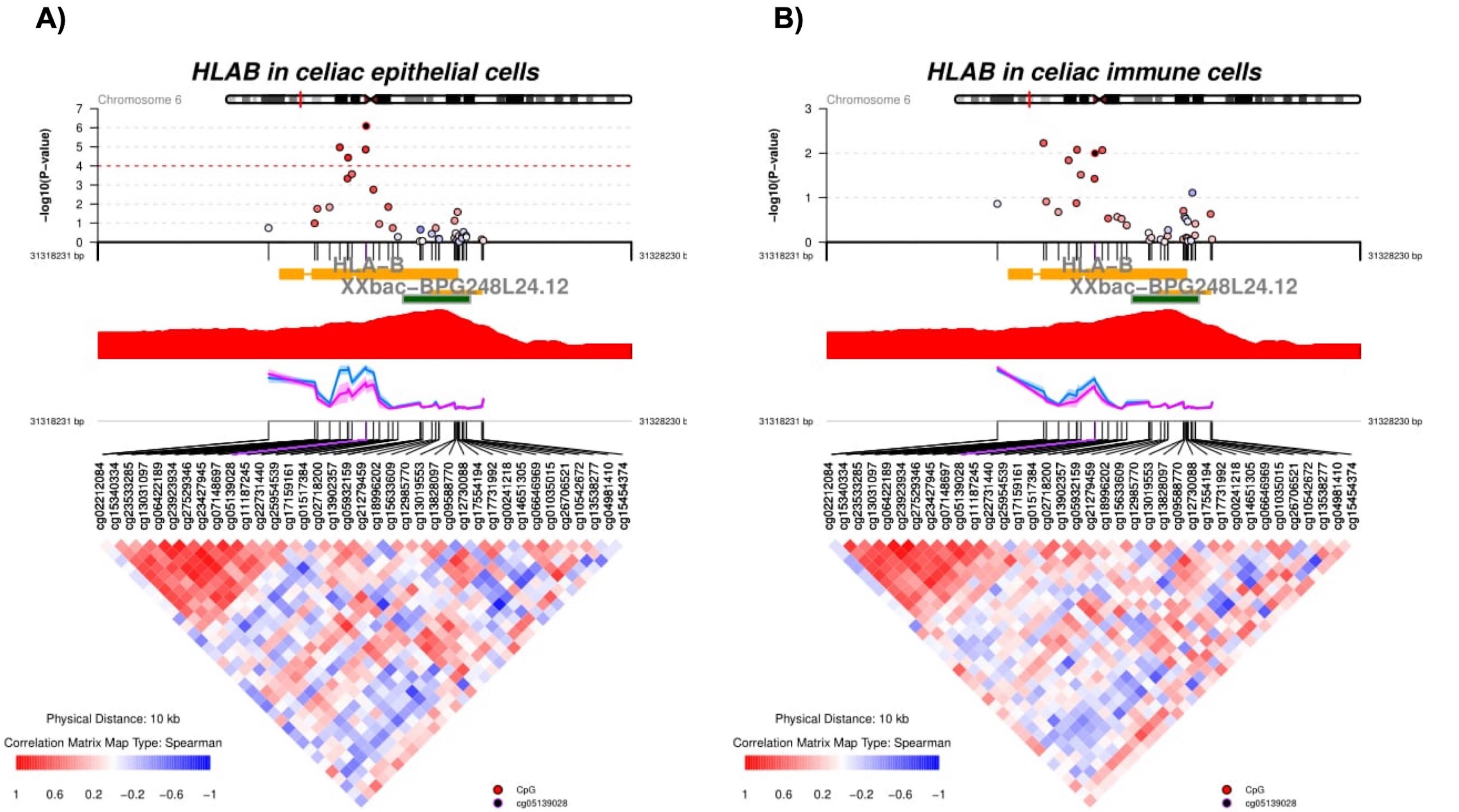
A)
HLAB in celiac epithelial cells
Chromosome 6
-log10(P-value)
31318231 bp
31328230 b
HLA-B
XXbac-BPG248L24.12
cg02212084 cg15340334 cg23533285 cg13031097 cg06422189 cg23923934 cg27529346 cg23427945 cg07148697 cg05139028 cg11187245 cg22731440 cg25954539 cg17159161 cg01517384 cg02718200 cg13902357 cg05932159 cg21279459 cg18996202 cg15633609 cg12985770 cg13019553 cg13828097 cg09588770 cg12730088 cg17554194 cg17731992 cg00241218 cg14651305 cg06646969 cg01035015 cg26706521 cg10542672 cg13538277 cg04981410 cg15454374
Physical Distance: 10 kb
Correlation Matrix Map Type: Spearman
1 0.6 0.2 −0.2 −0.6 −1
CpG
cg05139028
B)
HLAB in celiac immune cells
Chromosome 6
-log10(P-value)
31318231 bp
31328230 bp
HLA-B
XXbac-BPG248L24.12
cg02212084 cg15340334 cg23533285 cg13031097 cg06422189 cg23923934 cg27529346 cg23427945 cg07148697 cg05139028 cg11187245 cg22731440 cg25954539 cg17159161 cg01517384 cg02718200 cg13902357 cg05932159 cg21279459 cg18996202 cg15633609 cg12985770 cg13019553 cg13828097 cg09588770 cg12730088 cg17554194 cg17731992 cg00241218 cg14651305 cg06646969 cg01035015 cg26706521 cg10542672 cg13538277 cg04981410 cg15454374
Physical Distance: 10 kb
Correlation Matrix Map Type: Spearman
1 0.6 0.2 −0.2 −0.6 −1
CpG
cg05139028

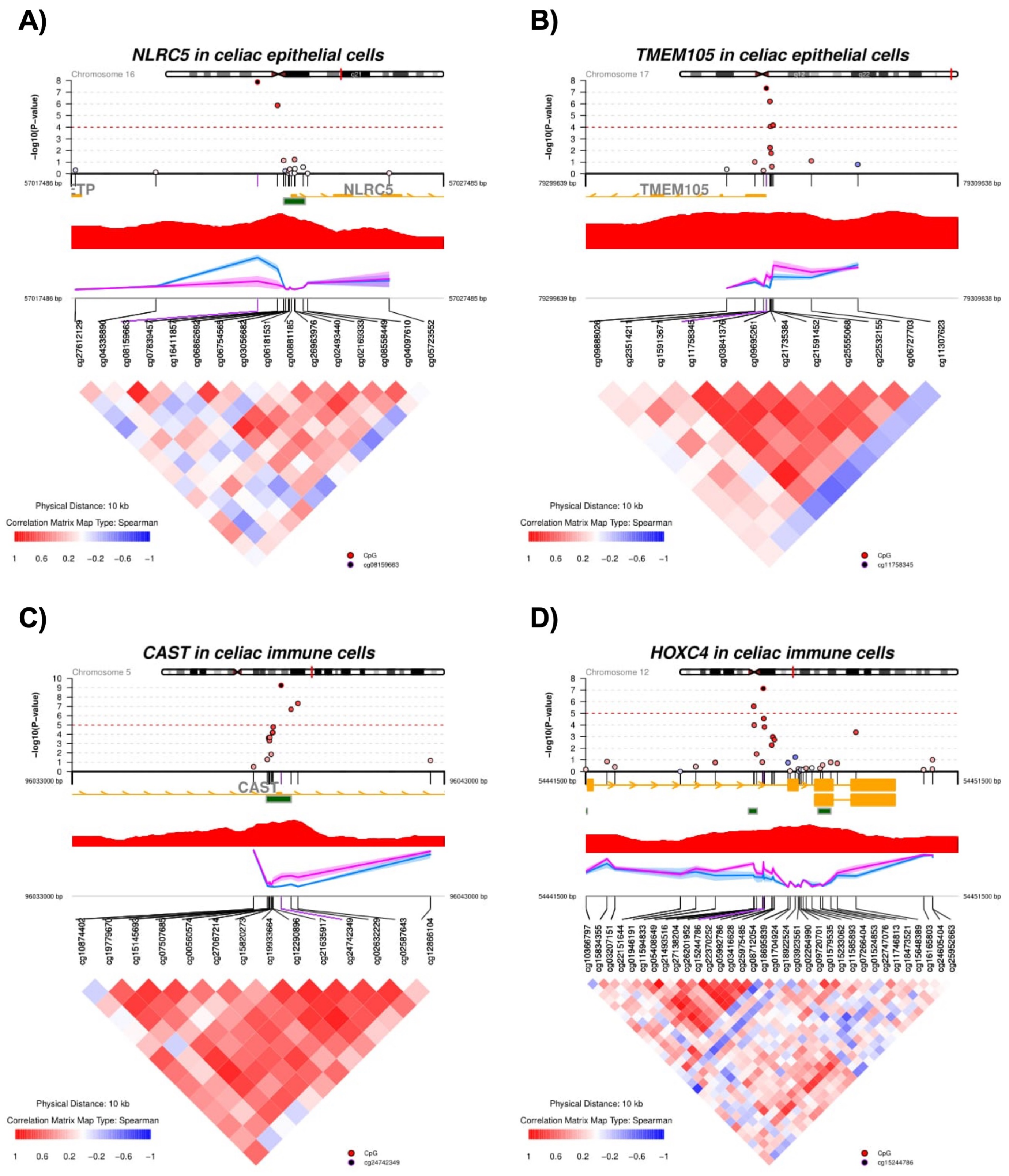
A)
NLRC5 in celiac epithelial cells
Chromosome 16
-log10(P-value)
57017486 bp
57027485 bp
NLRC5
Physical Distance: 10 kb
Correlation Matrix Map Type: Spearman
1 0.6 0.2 −0.2 −0.6 −1
CpG
cg08159663
B)
TMEM105 in celiac epithelial cells
Chromosome 17
-log10(P-value)
79299639 bp
79309638 bp
TMEM105
Physical Distance: 10 kb
Correlation Matrix Map Type: Spearman
1 0.6 0.2 −0.2 −0.6 −1
CpG
cg11758345
C)
CAST in celiac immune cells
Chromosome 5
-log10(P-value)
96033000 bp
96043000 bp
CAST
Physical Distance: 10 kb
Correlation Matrix Map Type: Spearman
1 0.6 0.2 −0.2 −0.6 −1
CpG
cg24742349
D)
HOXC4 in celiac immune cells
Chromosome 12
-log10(P-value)
54441500 bp
54451500 bp
Physical Distance: 10 kb
Correlation Matrix Map Type: Spearman
1 0.6 0.2 −0.2 −0.6 −1
CpG
cg15244786

S6 Fig

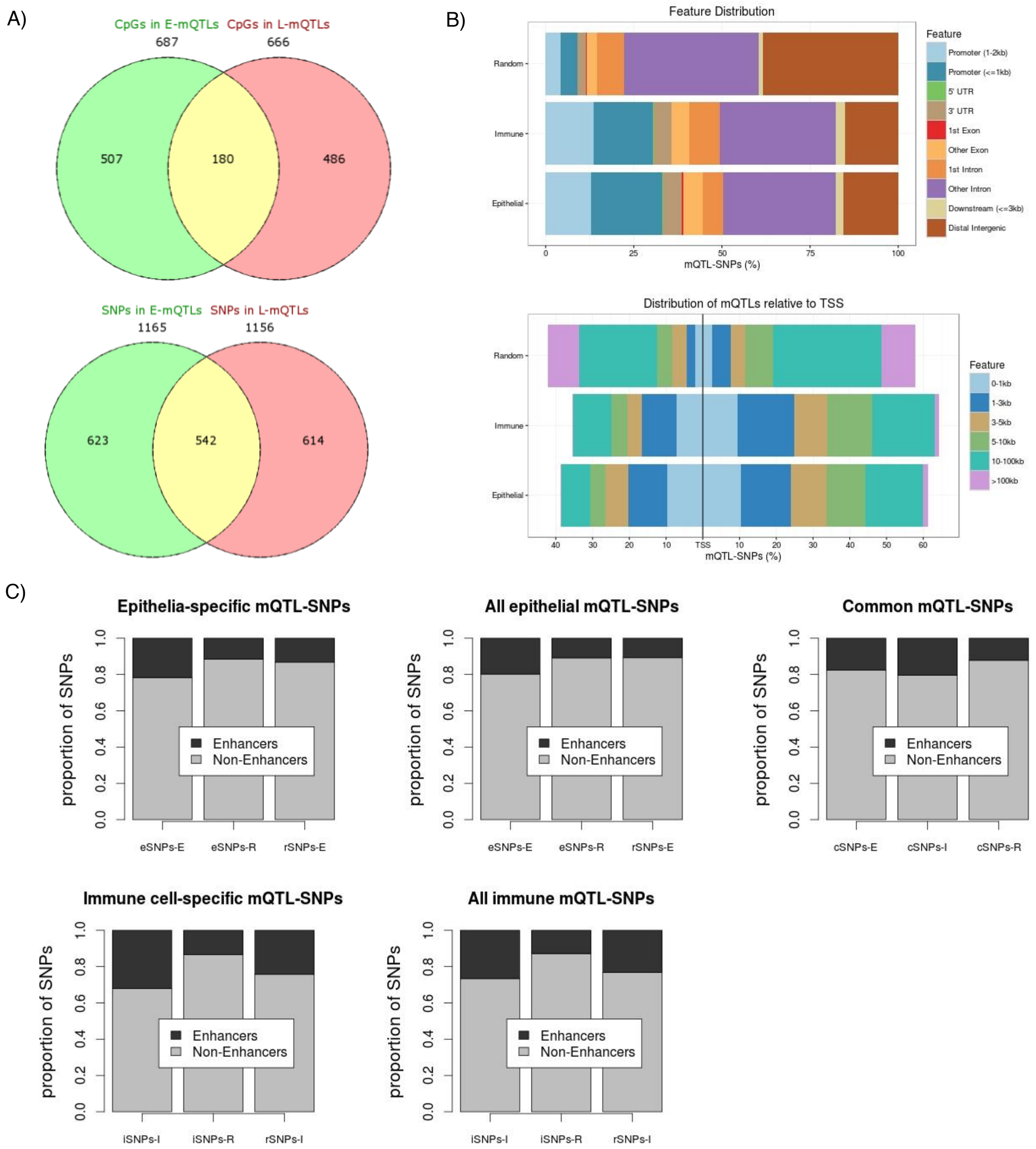
A)
CpGs in E-mQTLs
CpGs in L-mQTLs
687
666
507
180
486
SNPs in E-mQTLs
SNPs in L-mQTLs
1165
1156
623
542
614
B)
Feature Distribution
Random
Immune
Epithelial
0
25
50
75
100
mQTL-SNPs (%)
Feature
Promoter (1-2kb)
Promoter (<=1kb)
5' UTR
3' UTR
1st Exon
Other Exon
1st Intron
Other Intron
Downstream (<=3kb)
Distal Intergenic
Distribution of mQTLs relative to TSS
Random
Immune
Epithelial
40
30
20
10
TSS
10
20
30
40
50
60
mQTL-SNPs (%)
Feature
0-1kb
1-3kb
3-5kb
5-10kb
10-100kb
>100kb
C)
Epithelia-specific mQTL-SNPs
proportion of SNPs
0.0
0.2
0.4
0.6
0.8
1.0
Enhancers
Non-Enhancers
eSNPs-E
eSNPs-R
rSNPs-E
All epithelial mQTL-SNPs
proportion of SNPs
Enhancers
Non-Enhancers
eSNPs-E
eSNPs-R
rSNPs-E
Common mQTL-SNPs
proportion of SNPs
Enhancers
Non-Enhancers
cSNPs-E
cSNPs-I
cSNPs-R
Immune cell-specific mQTL-SNPs
proportion of SNPs
Enhancers
Non-Enhancers
iSNPs-I
iSNPs-R
rSNPs-I
All immune mQTL-SNPs
proportion of SNPs
Enhancers
Non-Enhancers
iSNPs-I
iSNPs-R
rSNPs-I

S7 Fig

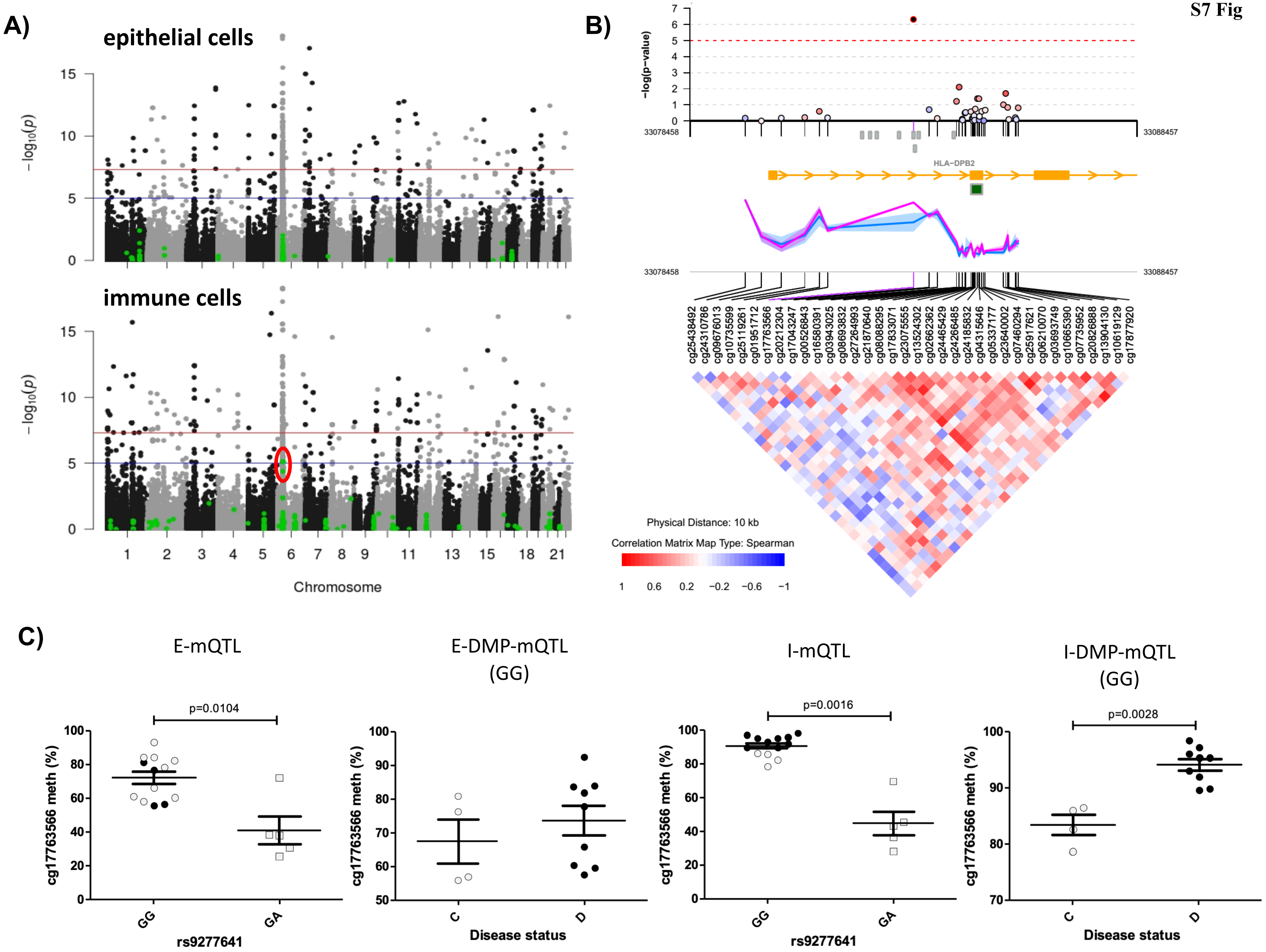

A)
epithelial cells
immune cells
−log10(p)
Chromosome
1 2 3 4 5 6 7 8 9 11 13 15 18 21
B)
−log(p−value)
33078458
33088457
HLA−DPB2
cg25438492
cg24310786
cg09676013
cg10735599
cg25119261
cg01951712
cg17763566
cg20212304
cg17043247
cg00526843
cg16580391
cg03943025
cg08693832
cg27264993
cg21870640
cg08088295
cg17833071
cg23075555
cg13524302
cg02662362
cg24465429
cg24266485
cg24185832
cg04315646
cg05337177
cg23640002
cg07460294
cg25917621
cg06210070
cg03693749
cg10665390
cg07735952
cg20826888
cg13904130
cg10619129
cg17877920
Physical Distance: 10 kb
Correlation Matrix Map Type: Spearman
1 0.6 0.2 −0.2 −0.6 −1
C)
E-mQTL
E-DMP-mQTL (GG)
I-mQTL
I-DMP-mQTL (GG)
p=0.0104
p=0.0016
p=0.0028
cg17763566 meth (%)
GG
GA
rs9277641
C
D
Disease status

Supplementary Table Headings

S1 Table. Characteristics of participants and experiments performed. All individuals were of Caucasian origin and recruited at the Pediatric Gastroenterology Unit of Cruces University Hospital between February 2014 and April 2015. All active CD patients were consuming gluten and had positive anti-transglutaminase autoantibodies and CD-compatible duodenal biopsy at the time the sample was taken. Controls were non-celiac individuals that underwent endoscopy for reasons other than CD, and did not present intestinal inflammation. GFD-treated CD patients had been diagnosed using the same criteria and biopsies were taken after 2 years on GFD, when they were asymptomatic, autoantibody-negative and presented a recovered duodenal biopsy.

S2 Table. Differentially Methylated Positions.

S3 Table. Gene expression data (normalized counts).

S4 Table. Differentially Variable and Methylated CpGs.

S5 Table. Differentially Methylated Regions.

S6 Table. Methylation Quantitative Trait *Loci*.

S7 Table. CD-associated non-HLA SNPs participating to cell type-specific mQTLs.

Biopsies for cell population studies

Sample ID	Sex	Group	Age (y)	Immunochip	CD45 (immune)		CD326 (epithelial)	
					RNAseq	Meth-450K	RNAseq	Meth-450K
214023	F	Active CD	11,2		X		X	
214024	F	Active CD	2,2				X	
214026	M	Control	9,0		X		X	
214027	M	Control	9,4	X		X	X	X
214028	M	Control	12,2		X		X	
214032	F	Control	8,5	X		X	X	X
214035	M	Active CD	6,0				X	
214041	M	Active CD	9,8				X	
214042	M	Control	12,4				X	
214046	F	Active CD	7,6		X		X	
214047	F	Control	12,9	X	X	X	X	X
214048	F	Control	5,9				X	
214052	M	Active CD	7,8	X		X*		X*
214053	F	Active CD	2,1	X	X	X	X	X
214054	F	Control	2,9				X	
214058	F	Active CD	7,6		X			
214059	M	Control	10,1				X	
214064	F	Control	11,2	X		X		X
214065	F	Active CD	8,3	X		X		X
214077	M	Control	5,4				X	
214089	F	Active CD	6,8	X		X	X	X
214091	F	Active CD	1,6				X	
214102	F	Active CD	2,2	X	X	X	X	X
214103	F	Active CD	1,6	X		X		X
214105	M	Active CD	13,9	X	X	X	X	X
214107	F	Control	12,1	X		X		X
214108	M	Active CD	2,2	X		X		X
214110	M	Control	14,2	X		X		X
214112	F	Control	7,3	X	X	X	X	X
214113	F	Control	3,0	X	X	X	X	X
214114	F	Control	8,1	X		X		X
215021	M	Active CD	4,7	X		X		X
215025	M	Active CD	8,3	X	X	X		X
215026	M	Control	7,0	X		X		X*

* excluded after Methylation QC

Whole biopsies for NGS

Sample ID	Sex	Group	Age (y)	Years on GFD
211022	F	GFD-treated CD	3,6	2,0
212062	F	GFD-treated CD	10,0	2,0
211027	F	GFD-treated CD	4,9	2,1
211014	F	GFD-treated CD	5,2	2,0
211087	F	GFD-treated CD	4,6	2,0
212045	M	GFD-treated CD	4,7	2,0
211021	F	GFD-treated CD	3,7	2,0
212101	F	Control	3,0	-
212108	M	Control	13,9	-
212106	M	Control	11,7	-
212109	F	Control	8,8	-
212095	M	Control	3,5	-
212111	M	Control	9,1	-
212103	F	Control	11,7	-
213004	F	Control	9,1	-
